# Direct Medical Cost of Influenza-Related Hospitalizations among Severe Acute Respiratory Infections Cases in Three Provinces in China

**DOI:** 10.1371/journal.pone.0063788

**Published:** 2013-05-22

**Authors:** Lei Zhou, Sujian Situ, Ting Huang, Shixiong Hu, Xianjun Wang, Xiaoping Zhu, Lidong Gao, Zhong Li, Ao Feng, Hui Jin, Shiyuan Wang, Qiru Su, Zhen Xu, Zijian Feng

**Affiliations:** 1 Public Health Emergency Center, Chinese Center for Disease Control and Prevention (China CDC), Beijing, China; 2 U.S. Centers for Disease Control and Prevention, Beijing, China; 3 Sichuan Provincial Center for Disease Control and Prevention, Chengdu, China; 4 Hunan Provincial Center for Disease Control and Prevention, Changsha, China; 5 Shandong Provincial Center for Disease Control and Prevention, Jinan, China; 6 Department of Epidemiology and Biostatistics, School of Public Health, Southeast University, Nanjing, China; 7 Key Laboratory of Surveillance and Early-warning on Infectious Disease, Division of Infectious Disease, Chinese Center for Disease Control and Prevention (China CDC), Beijing, China; Alberta Provincial Laboratory for Public Health/University of Alberta, Canada

## Abstract

**Background:**

Influenza-related hospitalizations impose a considerable economic and social burden. This study aimed to better understand the economic burden of influenza-related hospitalizations among patients in China in different age and risk categories.

**Methods:**

Laboratory-confirmed influenza-related hospitalizations between December 2009 and June 2011 from three hospitals participating in the Chinese Severe Acute Respiratory Infections (SARI) sentinel surveillance system were included in this study. Hospital billing data were collected from each hospital’s Hospital Information System (HIS) and divided into five cost categories. Demographic and clinical information was collected from medical records. Mean (range) and median (interquartile range [IQR]) costs were calculated and compared among children (≤15 years), adults (16–64 years) and elderly (≥65 years) groups. Factors influencing cost were analyzed.

**Results:**

A total of 106 laboratory-confirmed influenza-related hospitalizations were identified, 60% of which were children. The mean (range) direct medical cost was $1,797 ($80–$27,545) for all hospitalizations, and the median (IQR) direct medical cost was $231 ($164), $854 ($890), and $2,263 ($7,803) for children, adults, and elderly, respectively. Therapeutics and diagnostics were the two largest components of direct medical cost, comprising 57% and 23%, respectively. Cost of physician services was the lowest at less than 1%.

**Conclusion:**

Direct medical cost of influenza-related hospitalizations imposes a heavy burden on patients and their families in China. Further study is needed to provide more comprehensive evidence on the economic burden of influenza. Our study highlights the need to increase vaccination rate and develop targeted national preventive strategies.

## Introduction

Influenza viral infections are responsible for respiratory illnesses resulting in substantial morbidity and increased health care utilization and cost [1]. Influenza causes an annual 3 million cases of illness and 250,000 to 500,000 deaths globally [2]. Research has demonstrated substantial mortality associated with influenza-related illnesses in both temperate and subtropical areas of China [3]. The annual influenza-associated excess mortality was 18.0 (range: 10.9–32.7) and 11.3 (range: 7.3–17.8) deaths per 100,000 people in northern and southern cities, respectively [3]. Although only a small proportion of influenza infections required hospitalization, they imposed considerable economic and social burden [4], [5]. Understanding the cost of hospitalization is important in evaluating the economic burden of influenza and the impact of vaccination on a population scale.

Despite extensive research in developed countries, the socio-economic burden of laboratory-confirmed influenza in China has not been well-described. Currently, seasonal influenza vaccination in China is not covered by the national immunization program, instead requiring out-of-pocket payment. Influenza vaccination coverage remains low. A previous study examining production capacity, vaccine supply and annual sales from international and domestic vaccine manufacturers in China concluded the total available influenza vaccines could only cover 1.9% of the Chinese population [6]. Decision making on influenza prevention strategy requires scientific evidence on the cost-effectiveness of influenza vaccination. Data on the economic burden of laboratory-confirmed influenza provides important information for decision makers.

To better understand the economic burden of influenza hospitalization among different age groups in China, we conducted a study estimating the direct medical cost of influenza-related hospitalizations in three hospitals with Severe Acute Respiratory Infections (SARI) surveillance systems. SARI surveillance identified SARI cases in all ages, among which those with laboratory-confirmed influenza infection were recruited. We collected detailed cost data for cases from December 2009 to June 2011 and converted them to 2010 U.S. dollars (USD) [7]. We also identified factors resulted in high-cost hospitalizations.

## Materials and Methods

### Design, Setting and Patients

World Health Organization (WHO) recommended hospital-based surveillance of SARI be used to monitor severe diseases caused by influenza [8]. The Chinese SARI sentinel surveillance network was established during the 2009 H1N1 pandemic to monitor influenza-related diseases, identify possible risk factors for severe disease, and determine the etiological characteristics of influenza virus circulating in China [9]. There are 10 SARI sentinel hospitals in China. They are large-scale class III hospitals with special climatic features, characteristics of influenza epidemic, geographical representation, and previous work quality, as previously described [9].

The SARI case definition varies by age. For patients older than 5 years of age, clinical manifestations include acute illness onset, fever (≥38°C), cough or sore throat and breathing difficulty or tachypnea (≥25 breaths/minute). For patients 5 years of age or younger, clinical manifestations include acute illness onset, fever (≥38°C), cough or sore throat and one or more of the following symptoms: tachypnea [>60 breaths/minute (age <2 months), >50 breaths/minute (2–11 months), >40 breaths/minute (12 months to 5 years)], abnormal breath sounds on auscultation (e.g., rales, rhonchi, wheezing, dullness), and other general precarious signs, such as severe vomiting. Patients who met the SARI definitions were eligible for study enrollment. Nasopharyngeal and throat swabs were both collected and sent to one of the three influenza network laboratories to test for influenza viruses using reverse transcription-polymerase chain reaction and virus isolation [9]. Laboratory staff at these three sites were trained and accredited by staff at the National Influenza Center, Chinese Center for Disease Control and Prevention (China CDC). A patient was considered to have laboratory-confirmed influenza infection if at least one of the two test results was positive.

This study was conducted independent of routine SARI sentinel surveillance from January to June 2011 in three SARI sentinel surveillance hospitals in the provincial capitals of Sichuan (Hospital A), Hunan (Hospital B), and Shandong (Hospital C) with approximately 900 to 1000 beds per hospital. These three hospitals represented regions (western, central, eastern) with different levels of economic development. Among SARI cases reported from December 2009 to December 2010, we retrospectively included all hospitalized individuals with laboratory-confirmed influenza-related infections. We also prospectively recruited cases admitted from January to June 2011.

### Data Sources

We queried the Hospital Information System (HIS) to obtain hospital billing data for each influenza-related hospitalization case. The hospital costs were divided into five general categories: diagnostics, therapeutics, room and supplies, physician services, and nursing services. The division of these five categories was consistent with the study conducted in Children’s Hospital of Philadelphia [10]. The diagnostics category contained clinical examination and laboratory testing subcategories. Clinical examination included radiologic examination such as X-ray, B-ultrasound, electrocardiogram (ECG), computed tomography (CT), and magnetic resonance imaging (MRI). The therapeutics category included antiviral and antibiotics subcategories. We did not analyze detailed cost of the other therapeutics such as surgery, blood transfusion, and oxygen therapy due to a lack of specific data. Cost-to-charge ratios were not available from the hospitals, so it was assumed that charges were equivalent to costs. Costs were converted to 2010 USD values using an exchange rate of 1 USD for 6.67 Chinese Yuan (CNY) [7].

We collected clinical information on each influenza-related hospitalization by reviewing medical records including clinical and laboratory examinations; antiviral treatment; clinical complications such as pneumonia, respiratory failure, acute respiratory distress syndrome, heart failure, renal and hepatic dysfunction, disseminated intravascular coagulation and septic shock; clinical outcomes; and length of hospital stay. We also reviewed medical records for underlying medical conditions listed in the China Seasonal Influenza Vaccination Guidelines including chronic pulmonary (including asthma), chronic cardiovascular (except isolated hypertension), renal, hepatic, neurological, hematologic, and metabolic (including diabetes mellitus) disorders [11]. We compared collected information with SARI surveillance database and hospital records to confirm data accuracy.

### Analysis

Cases were divided into three age groups: children aged ≤15 years, adults aged 16 to 64 years, and elderly aged ≥65 years. In each age group, patients with underlying medical conditions were defined as high risk and patients without underlying medical conditions as low risk. In the overall group, we calculated the cost mean and range. In the subgroups we calculated cost and length of hospital stay (days) medians and interquartile ranges (IQR). Since hospital costs were highly skewed, we used Kruskal-Wallis tests to compare median cost across age groups and length of hospital stay (days) across risk subgroups. All statistical calculations were performed using SAS 9.1 (SAS Institute Inc, Cary, NC).

### Ethics Statement

This study was reviewed and approved by the Institute Review Board, Chinese Center for Disease Control and Prevention with registration with the Office for Human Research Protections (IRB 00005183, 2012). China CDC has a US Federal Wide Assurance (FWA 00002896). As a study on medical record data with no patients contact and no collection of personal data, the Chinese Center for Disease Control and Prevention Institute Review Board waived the need for written informed consent from the participants.

## Results

We retrospectively included 71 influenza-related hospitalizations among 560 SARI cases reported between December 2009 and December 2010. We prospectively recruited 35 influenza-related inpatients among 300 SARI cases reported from January to June 2011. Of the 106 total laboratory-confirmed influenza-related hospitalizations, 60% (64/106) were children younger than 15 years old and 21% (22/106) had underlying medical conditions (Table 1). The proportion of patients with underlying medical conditions was 2%, 44%, and 58% for children, adults, and elderly, respectively. More than 60% (64/106) of influenza patients had complications during hospitalization; most frequently among the elderly (79%). However, there was no statistically significant difference across the age groups. Although 61% (65/106) of influenza patients received antiviral treatment, only 32% of the elderly received antivirals, significantly lower than the other age groups (p<0.05). All patients, regardless of age, received antibiotic treatment. Nearly half (44%) of the children received radiological examination, significantly lower than the overall (61%) and the other two age groups (p<0.05). The median (IQR) length of hospital stay was 6 (6) days. The elderly group had longer length of hospital stay (14 days) than the other age groups (p<0.05).

**Table 1:** General characteristics of study groups.

Characteristics	Overall (N = 106)	Children (n = 64)	Adults (n = 23)	Elderly (n = 19)
Median age (IQR)	9 (46)	4 (5)	48 (29)	73 (11)
Male (%)	58 (54.7)	35 (64.7)	10 (43.5)	13 (68.4)
Underlying medical conditions (%)*	22 (20.8)	1 (1.6)	10 (43.5)	11 (57.9)
Complications (%)	64 (60.4)	36 (56.3)	13 (56.5)	15 (78.9)
Radiology (%)*	65 (61.3)	28 (43.8)	18 (78.3)	19 (100.0)
Routine blood test (%)	95 (89.6)	54 (84.4)	23 (100.0)	18 (94.7)
Blood biochemical test (%)	96 (90.6)	56 (87.5)	22 (95.7)	18 (94.7)
Sputum test (%)	10 (9.4)	8 (12.5)	1 (4.3)	1 (5.3)
Immunology test (%)	13 (12.3)	5 (7.8)	4 (17.4)	4 (21.1)
Antiviral treatment (%)*	65 (61.3)	47 (73.4)	12 (52.2)	6 (31.6)
Antibiotic treatment (%)	106 (100.0)	64 (100.0)	23 (100.0)	19 (100.0)
Median days of hospitalization (IQR)†	6 (6)	6 (4)	6 (9)	14 (23)
High risk group‡	10 (23)	7(n/a)§	8 (9)	11 (48)
Low risk group†	6 (6)	6 (4)	6 (10)	16 (11)

IQR: interquartile range.

* Statistical significant difference (p<0.05) was observed among three age groups using chi-square test;

† Statistical significant difference (p<0.05) was observed among three age groups using Kruskal-Wallis test;

‡ High risk group: Patients with underlying medical conditions; low risk group: patients without underlying medical conditions;

§ Only one case, thus no IQR applicable.

As shown in Table 2, the mean total direct medical cost of hospitalization was $1,797 with a skewed distribution (range: $80–$27,545). For children, adults, and elderly, the median total direct medical cost (IQR) was $231 ($164), $854 ($890), and $2,263 ($7,803), respectively. Comparing risk groups in the overall population and each age group, the high risk groups had higher costs. Overall, the total direct medical cost incurred by the patients with high risk (with underlying medical conditions) was 6 fold of the cost incurred by low risk patients (without underlying medical conditions). With the exception of antiviral cost, the cost of the other categories or subcategories for the high risk group was approximately 2–13 fold of the cost for the low risk group (p<0.05). For adults, the total cost, therapeutics, and antibiotics cost of the high risk group was approximately 2–3 fold of that of the low risk group (p<0.05). Nursing cost of the high risk elderly group was 3 fold of that of the low risk group (p<0.05). Higher costs were also found in the elderly group in the categories of total cost, diagnostics and therapeutics cost (Table 2). In our study, we had only one child with underlying medical conditions, thus precluding any comparisons from being drawn from this age group.

**Table 2:** Total and five categories of direct medical cost, median (IQR), of influenza-related hospitalizations by age and risk groups.

Cost Items	Overall†			Children		Adults‡		Elderly§	
	Total (n = 106)	HR* (n = 22)	LR* (n = 84)	HR (n = 1)	LR (n = 63)	HR (n = 10)	LR (n = 13)	HR (n = 11)	LR (n = 8)
Total cost#	317 (721)	1,554 (6,610)	264 (254)	260	271 (181)	1,148 (1,051)	525 (631)	2,340 (23,080)	1,295 (2,553)
Diagnostics‖	73 (215)	345 (430)	52 (79)	37	46 (28)	232 (399)	172 (205)	643 (1,788)	358 (241)
Examination** ^‖^	8 (68)	90 (250)	7 (23)	0	4 (8)	21 (86)	75 (106)	175 (398)	85 (71)
Laboratory testing‖	63 (151)	259 (305)	49 (55)	37	43 (29)	224 (214)	123 (107)	395 (1,625)	280 (129)
Therapeutics#	181 (450)	1,316 (5,997)	151 (158)	176	168 (106)	796 (1,059)	257 (366)	1,816 (16,432)	933 (2,367)
Antiviral#	0 (1)	0 (25)	0 (1)	0	0 (2)	0 (0)	0 (1)	0 (26)	0 (0)
Antibiotics#	78 (185)	283 (644)	66 (61)	35	61 (52)	311 (324)	103 (127)	303 (4,350)	535 (830)
Room and supplies	30 (42)	55 (124)	26 (33)	27	24 (26)	41 (52)	41(63)	81 (449)	75 (169)
Physician services	3 (6)	6 (7)	2 (6)	7	1 (5)	5 (5)	1 (6)	8 (14)	7 (8)
Nursing	11 (12)	31 (91)	9 (9)	13	8 (9)	12 (8)	6 (9)	52 (805)	17 (17)

* HR: high risk; LR: low risk.

** Examination includes radiologic examinations such as X-ray, B-ultrasound, ECG, CT, and MRI.

† Statistically significant difference (p<0.05) of total cost, diagnostics, examination, laboratory testing, therapeutics, antibiotics, room cost and supplies, physician services and nursing was observed between HR and LR groups using Kruskal-Wallis test;

‡ Statistically significant difference (p<0.05) of total cost, therapeutics and antibiotics was observed between HR and LR groups using Kruskal-Wallis test;

§ Statistically significant difference (p<0.05) of nursing was observed between HR and LR groups using a Kruskal-Wallis test;

# Statistically significant difference (p<0.05) was observed among three LR age groups using a Kruskal-Wallis test;

‖ Statistically significant difference (*p*<0.05) was observed among three HR age groups using a Kruskal-Wallis test, also among three LR age groups.

Comparing patients of different ages in the same risk category, elderly had a higher cost than other age groups. Among low risk, the total direct medical cost in the elderly was 5 and 2 times the cost for children and adults, respectively (p<0.05). Similar trends were observed for clinical examination and laboratory testing cost across the three age groups (p<0.05). Therapeutics cost in the elderly was also significantly higher than the other age groups (p<0.05). Among the high risk, diagnostics cost in the elderly was 3 times the cost in adults (p<0.05).

**Figure 1:**
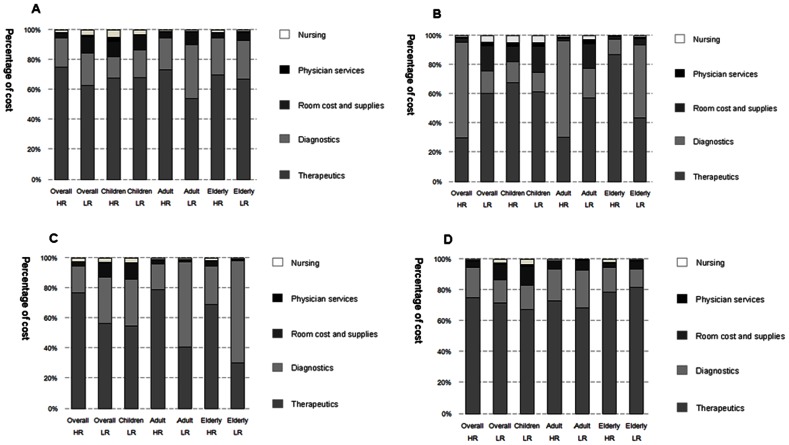
Hospitalization costs of influenza-related illnesses in three provincial hospitals in China. Individual costs of five categories (therapeutics, diagnostics, room and supplies, physician services, and nursing) for all patients from three hospitals (Panel A), patients from Hospital A in Sichuan (Panel B), patients from Hospital B in Hunan (Panel C) and patients from Hospital C in Shandong (Panel D) are shown. The age groups are children (≤15 years), adults (16–64 years), and elderly (≥65 years). HR: high risk; LR: low risk.

Regardless of age group or underlying medical conditions, therapeutics and diagnostics were the two largest components of direct medical cost of influenza-related hospitalizations, accounting for of 57% and 23%, respectively (Table 2 and Figure 1). In therapeutics, antiviral cost across age and risk groups was extremely low while antibiotic cost (overall median: $78, IQR: $185) was high. Clinical laboratory testing cost was the majority expenditure (∼84%) in the diagnostics cost, regardless of age group or underlying medical conditions. The clinical laboratory testing cost was much higher than the clinical examination cost (Table 2). Nursing and physician services costs were the lowest in the five categories: 3% and less than 1%, respectively.

Across hospitals, the proportion of each cost category varied. In Hospital A in Sichuan province, diagnostics constituted more than 50% of the total direct medical cost among high risk adult and overall groups. Among the high risk elderly group, the majority of cost was therapeutics while in the low risk elderly group, diagnostics and therapeutics accounted for nearly 50% each of total cost. In Hospital B in Henan province, therapeutics constituted the majority (>77%) of the total direct medical cost in overall high risk population. Therapeutics accounted for a higher proportion of total cost in Hospital C in Shandong province.

## Discussion

This is the first study on the direct medical cost of influenza-related hospitalizations in China. We found the mean cost of influenza-related hospitalization of 6 days (median: 6, IQR: 6) was $1,797. In contrast, the cost of outpatient influenza-like illness (ILI) visits was 71 fold lower ($25.2) in Guangdong province in southern China in 2007 [12]. This suggests that the direct medical costs of influenza-related hospitalization may be a greater source of economic burden to patients and their families than outpatient ILI.

The median direct medical cost of influenza-related hospitalization for children was $231 in our study, much lower than the corresponding cost in the United States ($3,366–$19,444) [10], [13], [14]. This may be due to different levels of economic development and medical care systems. A study analyzing data from 2005 to 2009 in a children’s hospital in Suzhou, Jiangsu province in eastern China, found the mean cost of influenza-related child hospitalizations was $624 [15]. Since it was a specialized hospital and the children had a higher proportion of complications (∼80%) than those in our study (∼60%), the children in the other study may have been sicker and required more expensive care than our population. However, the length of hospital stay was similar in the two studies (7 days in Suzhou. 6 days in this study). Considering the varied cost of living and the diversity of medical care service utilization, the differences observed in these two reports are not unexpected.

Hospitalization due to influenza-related illness incurred a high cost to patients. The overall direct medical cost ($1,797) was 41% of the Chinese annual gross domestic product (GDP) per capita ($4,433) in 2010 [16]. In contrast, hospitalization cost for the same illness ($590) in Hong Kong constituted 1.9% of the GDP per capita ($31,758) in 2010 [16], [17]. The cost of hospitalizing children ($231) was less than for adults or elderly. It constituted 5.2% of the annual GDP per capita. This figure was comparable to the 8.8% ($4,129/$46,702) in a report on children from the U.S. [13], [16].

Underlying medical conditions contributed to a 2–3 fold increase in hospitalization costs for adults and elderly. Annual influenza vaccination is the most effective method for preventing influenza viral infection and its complications [18]. Broader influenza vaccination coverage could decrease hospitalizations. Our findings support a targeted preventive vaccination strategy, focusing on high risk populations with underlying medical conditions in order to reduce economic burden to the society.

In agreement with a previous study [15], we found the majority of direct medical cost in our study hospitals was due to therapeutic treatment (57%) and diagnostic testing (23%). Only a small proportion was incurred through professional services such as physician services (<1%) and nursing (3%). This is drastically different from findings in the U.S., where the majority of hospitalization cost is from room charges and supplies (64%) [10].

Our findings also suggest that standardized clinical management could be optimized to properly guide physicians’ diagnostic testing use and treatment decisions. Laboratory testing was the major diagnostics expenditure in for all three age groups, regardless of underlying medical conditions. Clinical laboratory testing accounted for most of influenza-related hospitalization expenditures because of the high amount of laboratory testing especially the routine blood tests (90%) and blood biochemical tests (91%). Only a small proportion of patients received sputum tests (9%) and immunologic tests (12%) to confirm etiology. Another finding was the low use of antivirals which could be either due to an absence of medicines in the hospital or physician treatment preferences. However, the fact that all patients received antibiotics treatment indicated a potential problem of antibiotic overuse. The Ministry of Health recognized the need to optimize the practices and issued clinical management guidelines in October 2012 [19]. Future studies should investigate the impact of these guidelines on patient treatment, health outcomes and cost.

Our findings should be interpreted given the limitations of the study. We relied on the SARI surveillance system to identify cases; however some patients might not be captured by the system due to suboptimal implementation of the SARI protocol. Since our data was drawn from provincial hospitals, our study might bias toward the selection of relatively more severe cases, who attended provincial rather than more local hospitals. Despite collecting data over two and a half influenza seasons with hospitals in three locations, the size of the study population was small (n = 106) and may not be generalizable to the rest of China. The expenditure and cost composition in more developed cities, such as Beijing and Shanghai may be different from those found in other areas. It is likely that physicians may provide different services based on health insurance status of the patients, but due to inadequate information on health insurance, we were unable to assess its impact.

This study focused on direct medical cost of influenza-related hospitalizations among SARI cases. Future work should include direct non-medical cost and indirect costs including transportation and lost productivity. To accurately measure the annual social and economic burden and impact attributed to influenza infections in China, further investigation is needed on the cost of influenza-related outpatient care and non-SARI influenza-related hospitalizations.

### Conclusions

Direct medical cost of laboratory-confirmed influenza-related hospitalizations in three Chinese provinces indicates a heavy economic burden on patients and their families. This burden is increased if the patient has underlying medical conditions. Further study in more diverse hospitals is needed to yield more comprehensive evidence on social and economic burden. Our study highlights the need to develop targeted prevention strategies and medications to reduce economic burden.
